# Localization and functional characterization of the pathogenesis-related proteins Rbe1p and Rbt4p in *Candida albicans*

**DOI:** 10.1371/journal.pone.0201932

**Published:** 2018-08-06

**Authors:** Yannick Bantel, Rabih Darwiche, Steffen Rupp, Roger Schneiter, Kai Sohn

**Affiliations:** 1 Institute of Interfacial Process Engineering and Plasma Technology, University of Stuttgart, Stuttgart, Germany; 2 Department of Biological Chemistry and Molecular Pharmacology, Harvard Medical School, Boston, Massachusetts, United States of America; 3 Department of Molecular Biotechnology, Fraunhofer IGB, Stuttgart, Germany; 4 Department of Biology, University of Fribourg, Fribourg, Switzerland; University of California Los Angeles David Geffen School of Medicine, UNITED STATES

## Abstract

Members of the Cysteine-rich secretory protein, Antigen 5 and Pathogenesis-related 1 (CAP) protein superfamily are important virulence factors in fungi but remain poorly characterized on molecular level. Here, we investigate the cellular localization and molecular function of Rbe1p and Rbt4p, two CAP family members from the human pathogen *Candida albicans*. We unexpectedly found that Rbe1p localizes to budding sites of yeast cells in a disulfide bond-dependent manner. Furthermore, we show that Rbe1p and Rbt4p bind free cholesterol *in vitro* and export cholesteryl acetate *in vivo*. These findings suggest a previously undescribed role for Rbe1p in cell wall-associated processes and a possible connection between the virulence attributes of fungal CAP proteins and sterol binding.

## Introduction

Proteins of the CAP (Cysteine-rich secretory proteins, Antigen 5, Pathogenesis-related 1) superfamily show high evolutionary conservation and are widespread throughout prokaryotic and eukaryotic phyla [1]. CAP family members are associated with diverse biological processes, including immune defense, venom toxicity, reproduction and cancer development [1]. Despite their functional and evolutionary diversity, their molecular mode of action remains largely elusive. The characterizing feature of these proteins is the conserved 17- to 21-kDa CAP domain, which adopts a unique α-β-α sandwich fold, stabilized by disulfide bonds [1, 2]. Additional N- and C-terminal extensions with low sequence similarity are common among CAP family members and most proteins contain a signal peptide for protein secretion to the extracellular space [1].

The human fungal pathogen *Candida albicans* encodes five CAP proteins and two of them, Rbe1p and Rbt4p, have been shown to be important virulence factors [3–5]. In mass spectrometric analyses, Rbe1p peptides were exclusively found in the supernatant of yeast cells, while Rbt4p peptides were enriched under hyphal growth conditions [3, 6]. Deletion of *RBE1* and *RBT4* in a clinical *C*. *albicans* isolate leads to a virulence defect in a mouse model for disseminated candidiasis and fungal hypersensitivity towards the attack by human polymorphonuclear leucocytes [3]. Besides modulating host-pathogen interactions, *RBE1* and *RBT4* deletions do not have any obvious influence on morphological, metabolic or stress-related features of *C*. *albicans* [3]. Therefore, biochemical and structural analysis of CAP proteins from other organisms might help our understanding of Rbe1p and Rbt4p function.

Recent investigations identified lipid binding and export as a potential molecular function of the CAP domain. The CAP superfamily members Pry1p and Pry2p from *Saccharomyces cerevisiae* have been shown to bind sterols and fatty acids *in vitro* and to export intracellularly accumulated cholesteryl acetate and fatty acids *in vivo* [7–10]. Mutational analysis of *S*. *cerevisiae* Pry1p revealed that the caveolin-binding motif (CBM), a flexible loop containing aromatic amino acid residues, is required for sterol binding [11].

Finding the link between the virulence phenotype and the molecular function of Rbe1p and Rbt4p has remained a challenge, especially because no functional or structural data on protein level is available yet. Here, we characterize Rbe1p and Rbt4p on molecular level and describe a novel disulfide bond-dependent association of Rbe1p with the yeast cell wall. We furthermore link Rbe1p and Rbt4p function to sterol binding and export.

## Materials and methods

### Media and growth conditions

*C*. *albicans* strains were routinely grown in YPD (1% yeast extract, 2% Bacto peptone, 2% glucose) or SC medium (0.17% yeast nitrogen base, 0.5% ammonium sulfate, and 2% glucose, supplemented with amino acids and adjusted to pH 6.6 with 10 mM NaOH). For protein isolation or immunofluorescence experiments, YPD overnight cultures were diluted to an OD_600_ of 0.3 and grown in RPMI 1460 media (Gibco, Invitrogen) at 37°C to induce hyphal growth or in SC medium at 30°C to maintain yeast growth. For all experiments cells were grown to exponential phase.

*S*. *cerevisiae* mutant strains were cultivated in YPD media or minimal media (containing 0.67% yeast nitrogen base without amino acids, 0.73 g L^-1^ amino acids, and 2% glucose). Media supplemented with sterols contained 0.05 mg ml^-1^ Tween 80 and 20 μg ml^-1^ cholesterol (Sigma-Aldrich). To bypass heme deficiency, *hem1Δ* mutant cells were supplemented with 10 μg ml^-1^ delta-aminolevulinic acid.

A complete list of all the strains used in this study is contained in the supplementary material (S1 Table).

### Construction of recombinant strains

#### Candida albicans

Epitope-tagging of *RBE1* and *RBT4* in the *C*. *albicans* clinical isolate SC5314 [12] was done using the *SAT1* flipping method previously described by Reuss *et al* [13]. For this purpose, *RBE1* and *RBT4* open reading frames, excluding stop codons, were PCR amplified with additional upstream flanking regions using the primers listed in the supplementary material (S2 Table). Chromosomal integration of the constructs and excision of the *SAT1* cassette was done as previously described [3, 14].

#### S. cerevisiae

For recombinant expression of *RBE1* and *RBT4* in the *S*. *cerevisiae pry1Δpry2Δ* strain, endogenous *RBE1* and *RBT4* open reading frames, excluding signal peptides sequences, were PCR amplified using primers containing the pre-pro alpha factor signal sequence. The PCR products were cloned into plasmid pRS416 by homologous recombination and correct integration of the constructs was confirmed by colony PCR.

#### Escherichia coli

For heterologous expression of Rbe1p and Rbt4p in *E*. *coli*, *RBE1* and *RBT4* gene sequences were codon optimized (S7 Fig), chemically synthesized with additional XhoI and BamHI restriction sites and cloned into vector pET-19b(+) (GenScript, Piscataway, USA). Transformation of SHuffle® T7 Competent *E*. *coli* cells (New England Biolabs) with the plasmids was done using the method described by Chung *et al*. [15].

A list of all primer sequences and constructed plasmids is found in the supplementary information (S2 and S3 Tables).

### Protein isolation and purification

#### Candida albicans

To isolate proteins from the DTT-soluble cell wall fraction of *C*. *albicans*, cell pellets were resuspended in lysis buffer (50 mM Tris-HCl pH 7.5, 1x EDTA-free protease inhibitor cocktail (Complete; Roche) 1mM phenylmethyl-sulfonylfluoride) and lysed using bead beating or high-pressure homogenization (Emulsiflex B-15, Avestin). The remaining cell debris was washed once with lysis buffer and then twice in SH-reducing buffer (50 mM Tris-HCl pH 7.5, 100 mM DTT or alternatively 4% ß-mercaptoethanol). Wash fractions were pooled and analyzed directly by SDS-PAGE/Western blotting.

Concentration of culture supernatant was achieved using Amicon Ultra-15 centrifugal filter units, MWCO 10 kDa (Merck) or Centricon® Plus-70 centrifugal filter units (Merck) depending on the supernatant volume. Centrifugation speed and time were chosen according to the specifications of the manufacturer.

#### Escherichia coli

Expression of polyhistidine-tagged fusion proteins in SHuffle® T7 Competent *E*. *coli* cells was induced by lactose at 24°C overnight. Cells were harvested, lysed, and incubated with Ni-NTA beads (Qiagen) according to the manufacturer instructions; beads were washed, and proteins were eluted with imidazole. Protein concentration was determined by Lowry assay using Folin reagent and BSA as standard.

### SDS-PAGE and protein detection

Protein samples were separated on 6% SDS-PAGE according to the method of Laemmli [16]. For immundetection, proteins were transferred to a polyvinylidene difluoride membrane (Immobilon-P; Millipore) using a semidry transfer unit (Hoefer TE77X semidry transfer unit). Blocking of the membrane was carried out in PBS (pH 7.4) with 5% skim milk for 1 h at room temperature or overnight at 4°C. After a washing step of 10 min in PBS (pH 7.4) with 0.05% Tween 20, membranes were incubated with mouse monoclonal anti-V5 antibody (1:5.000; Clone SV5-PK1 Acris) in PBS (pH 7.4) with 0.05% Tween 20 and 0.5% BSA. Subsequent detection occurred via peroxidase-coupled sheep anti-mouse antibody (1:5.000; GE Healthcare) and ECL Plus chemiluminescence substrate (Pierce) using a LAS-1000 CCD camera (Fuji Photo Film).

### Indirect immunofluorescence and wheat germ agglutinin staining

*C*. *albicans* yeast cells were fixed directly in suspension for 1 h at 30°C by adding 37% formaldehyde solution to a final concentration of 3.7%. After fixation, cells were washed in phosphate-buffered saline (PBS; pH 7.4) and stained with 250 μg ml^-1^ FITC-conjugated wheat germ agglutinin (Sigma-Aldrich) for 30 min at room temperature. After washing of unbound FITC-WGA with PBS (pH 7.4), cells were immobilized on poly-L-lysine (0.1 mg ml^-1^, Sigma-Alrich) coated glass slides for 20 min at room temperature. Blocking was carried out in PBS (pH 7.4) with 2% bovine serum albumin for 1 h at room temperature or overnight at 4°C. For immunofluorescence, cells were stained with mouse monoclonal anti-V5 antibody (1:100; Clone SV5-PK1, Acris) in Dako antibody diluent (Agilent) for 1 h at room temperature and goat anti-mouse Alexa Fluor 555 antibody (1:400; Thermo Fisher) for 30 min at room temperature. Mowiol mounted slides were imaged using the 63x objective of a Zeiss Axio Observer Z1 microscope and *Axiovision* software.

### In vitro sterol-binding assay

The sterol-binding assay was performed as previously described by Im *et al*. [17]. For each binding reaction 100 pmol purified protein was incubated with 100–500 pmol [^3^H]-cholesterol in a final volume of 100 μl binding buffer (20 mM Tris, pH 7.5, 30 mM NaCl, 0.05% Triton X-100). After incubation for 90 min at 30°C, protein was separated from the unbound ligand by adsorption to Q Sepharose anion-exchange beads (GE Healthcare). The beads were washed with washing buffer (20 mM Tris, pH 7.5), proteins were eluted by the addition of 0.5 ml of elution buffer (20 mM Tris, pH 7.5, 1 M NaCl) and the bound [^3^H]-cholesterol was quantified by scintillation counting. For competition assays, 50 pmol or 500 pmol of unlabeled cholesterol were included in the binding reaction, together with 50 pmol of [^3^H]-cholesterol. To determine nonspecific binding, the binding reaction was performed in the absence of added protein. Data were analyzed using PRISM software (GraphPad).

### Yeast sterol export assay

Acetylation and export of sterols into the culture supernatant was examined as previously described using heme-deficient yeast cells lacking the sterol deacetylase *SAY1* [7, 18]. Mutant cells with or without plasmid pRS416-*RBE1* or pRS416-*RBT4* were cultivated in the presence of cholesterol/Tween-80-containing media and labeled with 0.025 μCi/ml [^14^C]-cholesterol (American Radiolabeled Chemicals). Cells were harvested by centrifugation, washed twice with synthetic complete media, diluted to an OD_600_ of 1 into fresh media containing non-radiolabeled cholesterol, and grown overnight. Afterwards lipids were extracted from the cell pellet and the culture supernatant using chloroform/methanol (1:1). Samples were dried and separated by thin layer chromatography plate using silica gel 60 plates (Merck) and petroleum ether/diethyl ether/acetic acid (70:30:2; per vol) as a solvent system. TLC plates were then exposed to phosphorimager screens and radiolabeled lipids were visualized using a phosphorimager (GE Healthcare).

### Sequence analysis and homology modelling

Multiple sequence alignments were generated using Clustal Omega [19] (www.ebi.ac.uk/Tools/msa/clustalo) using default settings. 3D structure models of Rbe1p and Rbt4p were constructed using the web-based I-Tasser server (www.zhanglab.ccmb.med.umich.edu/I-TASSER) [20]. For the homology modelling only the conserved CAP domain was used (Rbe1p: residues F123 –L271; Rbt4p: residues F211 –Q358). The resulting models with the highest C-scores, and therefore highest confidence, were used. PyMOL software was used to visualize molecular protein structures.

## Results

### Localization of Rbe1p and Rbt4p in *C*. *albicans*

A striking feature of *C*. *albicans* is its ability to grow either as an unicellular budding yeast or in filamentous pseudohyphal and hyphal forms. This morphological plasticity is an important virulence determinant, as the hyphal form holds a key role in the infection process [21]. We therefore analyzed the expression level and localization of Rbe1p and Rbt4p during yeast and hyphae growth conditions. To facilitate immunological detection, a V5/His6-tag was fused to the C-terminus of endogenous Rbe1p and Rbt4p.

**Fig 1:**
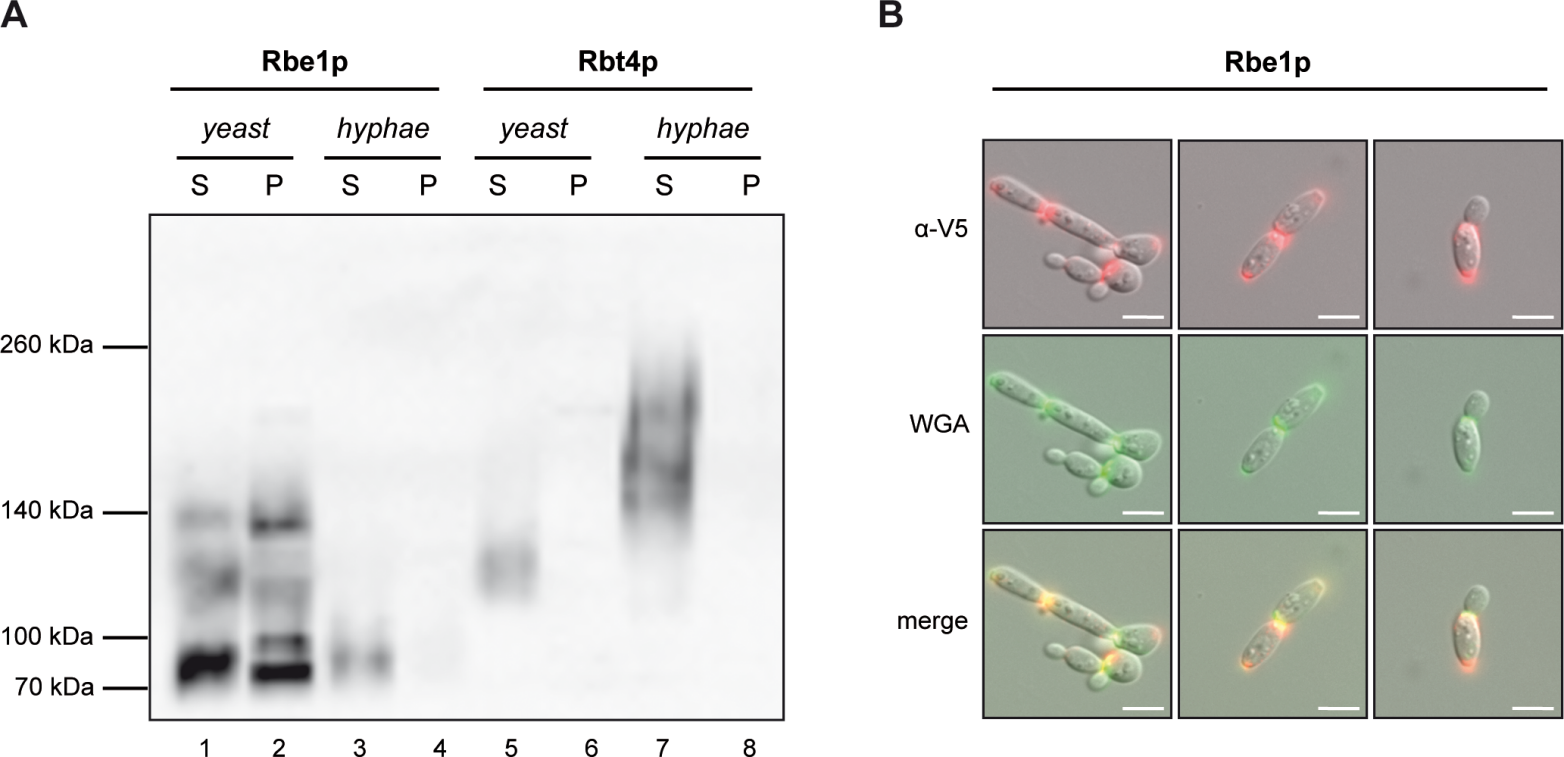
Expression and localization of Rbe1p and Rbt4p in *C*. *albicans*. **(A)** Supernatant (S) and DTT-sensitive cell wall fraction (P) from *C*. *albicans* yeast and hyphal cells were analyzed for the presence of V5-tagged Rbe1p and Rbt4p. An equal relative volume of each fraction was loaded in each lane and the proteins were detected by immunoblotting with a mouse anti-V5 antibody. **(B)** Localization of V5-tagged Rbe1p in *C*. *albicans* yeast cells was assessed using indirect immunofluorescence. Formaldehyde fixed cells were stained using a primary mouse anti-V5 antibody and a secondary Alexa Fluor 555 coupled goat anti-mouse antibody (red). Additionally, N-acetyl-glucosamine was co-stained with FITC-conjugated wheat germ agglutinin (WGA, green). Scale bar: 5 μm.

Western blotting of culture supernatant showed that both Rbe1p and Rbt4p migrated much slower than deduced from their primary sequence (Rbe1p~ 32 kDa; Rbt4p~ 40 kDa) (Fig 1A). Protein bands of varying molecular weight and intensity were detectable, which most likely emerge due to glycosylation and/or protein complex formation. Using chemical and enzymatic deglycosylation methods, we could indeed show that both Rbe1p and Rbt4p become O-glycosylated, while Rbe1p is additionally N-glycosylated (S1 Fig).

In the supernatant of yeast cells three main signals were detected for Rbe1p, a very prominent band at ~75 kDa and two weak and less defined bands at ~ 120 kDa and ~135 kDa (Fig 1A, lane 1), while only one faint band appeared for Rbt4p at ~120 kDa (Fig 1A, lane 5). In the supernatant of hyphal cells only a weak ~75 kDa band for Rbe1p was detectable (Fig 1A, lane 3), while a more intense and smeary Rbt4p signal was visible between ~140 kDa– 240 kDa (Fig 1A, lane 7). These growth condition dependent patterns indicate that besides differential expression, differential post-transcriptional modification seems to be an important feature of Rbe1p and Rbt4p. In agreement with previous mass spectrometric data [3], higher levels of Rbe1p were detected in the supernatant of yeast cells compared to hyphal cells, while Rbt4p showed the opposite expression pattern. As expected for proteins with a secretion signal, the levels of Rbe1p and Rbt4p in the cytoplasmic lysate were almost undetectable (S2 Fig).

Even though Rbe1p and Rbt4p lack potential GPI-anchor sites and transmembrane domains, attachment to the cell wall by other means is conceivable [3, 22]. Therefore, in addition to analyzing the culture supernatant for the presence of tagged Rbe1p and Rbt4p, we also included the disulfide-labile protein fraction of the cell wall in our analysis. Note that the comparability between the different samples on the western blot is justified by taking equal amounts of all fractions derived from the same number of cells cultured under identical conditions, avoiding the use of unreliable loading controls for secreted or cell wall proteins.

Surprisingly, treatment of yeast cell debris with disulfide-reducing agents like beta-mercaptoethanol or dithiothreitol (DTT) led to a strong release of tagged Rbe1p (Fig 1A, lane 2). This disulfide bond-dependent localization of Rbe1p seemed to be very specific as it was absent in hyphal cells (Fig 1A, lane 4) and not observed for tagged Rbt4p under identical growth conditions (Fig 1A, lane 6, 8). The amount of Rbe1p solubilized by DTT was comparable or even higher than the amount present in the supernatant. Furthermore, the band pattern corresponding to tagged Rbe1p differed considerably from the supernatant fraction; an additional signal at ~90 kDa was detected and the band at ~135 kDa was more intense. As dimerization has been observed for different CAP proteins [23–26], we wondered, whether the 135 kDa western blot signal might be an Rbe1p homodimer. Using V5-tag affinity purification and mass spectrometry, we primarily detected peptides corresponding to Rbe1p in the 135 kDa band, confirming that it is most likely an Rbe1p homodimer (S3 Fig and S4 Table). Remarkably, this dimerization is unusually stable as it resists the stringent denaturating conditions of an SDS-PAGE.

To gain more precise insights into the localization of Rbe1p in *C*. *albicans* yeast cells, we performed indirect immunofluorescence experiments. Fluorescence microscopy revealed a polar localization of Rbe1p in the yeast cell wall (Fig 1B). In accordance with the results from the western blot, no comparable fluorescence signal was present in hyphal cells or yeast cells expressing V5/His6-tagged Rbt4p (S4 Fig). As Rbe1p seemed to be enriched at budding sites (bud scars or birth scars) and these sites contain the N-acetyl-glucosamine polymer chitin, we used fluorescein labeled wheat germ agglutinin (FITC-WGA) for co-localization. WGA binds specifically to N-acetyl-glucosamine in bud scars but only weakly to chitin elsewhere in the cell wall [27]. Rbe1p showed stable co-localization at sites of chitin deposition in yeast cells, confirming its enrichment at budding sites (Fig 1B). General chitin localization or deposition did not seem to be influenced by Rbe1p, as an *rbe1Δ* deletion strain showed no difference in FITC-WGA staining compared to its parental strain (S5 Fig).

### Sterol binding and export function of Rbe1p and Rbt4p

**Fig 2:**
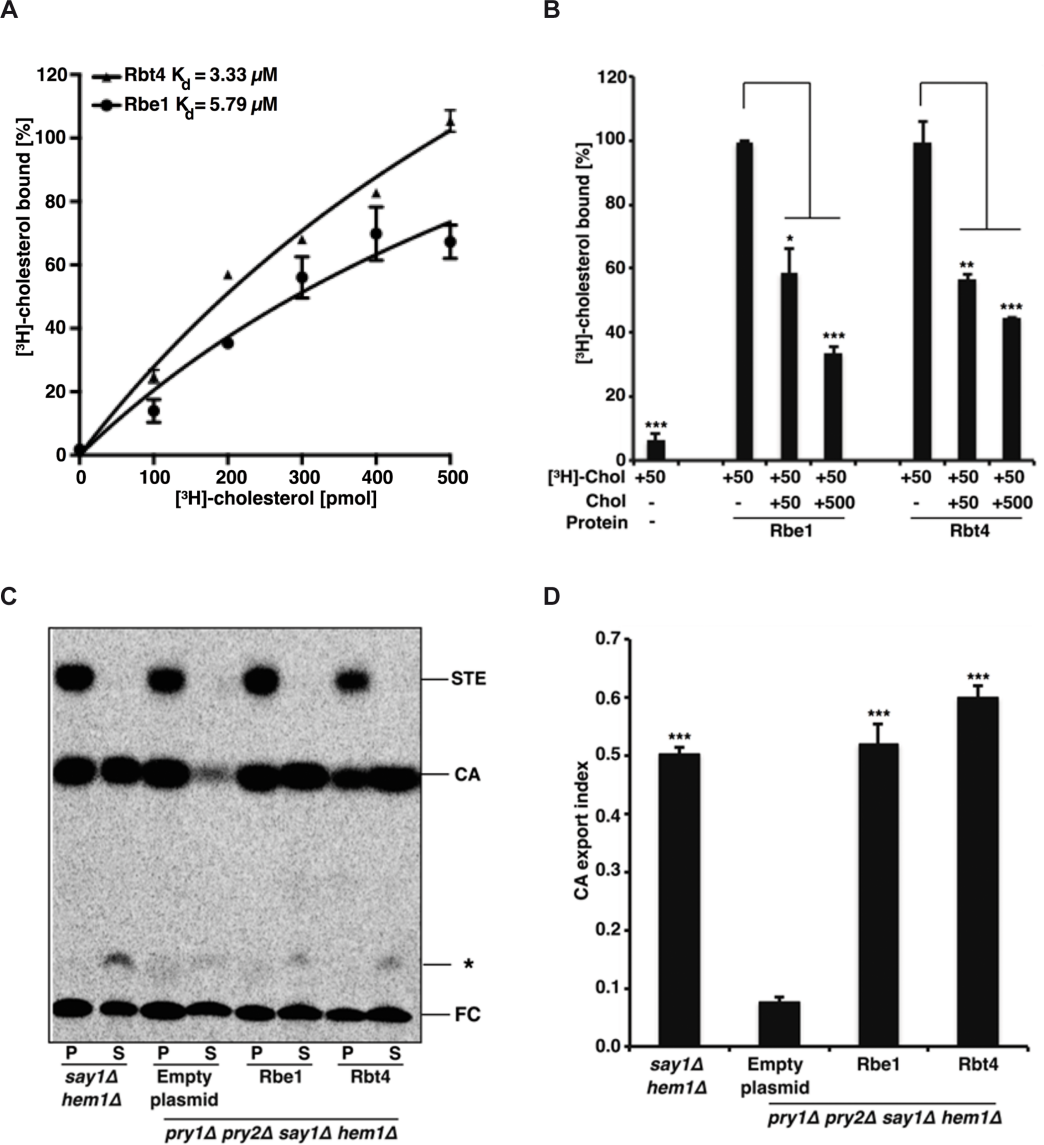
Rbe1p and Rbt4p bind cholesterol *in vitro* and rescue the sterol export defect of yeast cells lacking *PRY1* and *PRY2*. **(A)** Purified Rbe1p and Rbt4p bind cholesterol *in vitro*. Sterol binding was assessed using increasing amounts of [^3^H]-cholesterol (100–500 pmol) and 100 pmol of purified protein. Unbound radioligand was separated from proteins, and the bound radioligand was quantified by scintillation counting. Data represent the mean ± SD of four independent experiments. **(B)** Cholesterol binding specificity of Rbe1p and Rbt4p was assessed using 100 pmol purified protein incubated with 50 pmol of [^3^H]-cholesterol in the presence of 50 pmol or 500 pmol unlabeled cholesterol. Competition results are plotted relative to the ligand binding of Rbe1p and Rbt4p. Data represent the mean ± SD of three independent experiments. Statistical significance of data was analyzed by a multiple t-test. Asterisks denote statistical significance (* P < 0.05; ** P < 0.001; *** P < 0.0001). **(C)** Expression of *RBE1* or *RBT4* complements the sterol export defect of *S*. *cerevisiae* cells lacking endogenous CAP proteins. *pry1Δpry2Δsay1Δhem1Δ* cells containing either an empty plasmid or a plasmid carrying *RBE1* or *RBT4* were radiolabeled with [^14^C]-cholesterol and the extracted lipids from the cell pellet (P) and the culture supernatant (S) were separated by thin-layer chromatography. The positions of free cholesterol (FC), cholesteryl acetate (CA) and steryl esters (STE) are indicated on the right. The asterisk marks the position of an unidentified cholesterol derivative. **(D)** Export index indicating the relative fraction (exported CA/total CA) of cholesteryl acetate that is exported by the corresponding mutant strains. Data represent the mean ± SD of two independent experiments. Statistical significance of data was analyzed by a multiple t-test. Asterisks denote statistical significance (* P < 0.05; ** P < 0.001; *** P < 0.0001).

The ability to bind and export sterols has been experimentally confirmed for different CAP proteins [7, 9, 10]. Noteworthy, there are also examples of CAP proteins that do not show sterol binding but have evolved other ligand binding specificities [8, 28]. As binding and sequestering of host sterols might influence virulence attributes by compromising host cell membrane integrity or signal transduction, we wanted to investigate the ability of Rbe1p and Rbt4p to bind sterols. To assess sterol binding, we expressed codon-optimized His-tagged versions of Rbe1p and Rbt4p in *Escherichia coli* and purified them via affinity chromatography on nickel agarose beads. Purified protein was then used in an *in vitro* [^3^H]-cholesterol binding assay with increasing amounts of radioligand [29]. For the assay cholesterol was chosen over ergosterol, that represents the major sterol molecule in fungi, to enable the comparison with previous sterol binding studies of CAP proteins. Measuring radioactivity showed saturable binding of cholesterol by Rbe1p and Rbt4p with a dissociation constant K_D_ of ~5,78 μM and ~3,3 μM, respectively (Fig 2A). Binding specificity was further validated in a competitive binding assay with unlabeled cholesterol. Including equimolar or excessive amounts of unlabeled cholesterol in the binding reaction significantly decreased the amount of bound [^3^H]-cholesterol for both proteins (Fig 2B), indicating successful competition of unlabeled cholesterol with radioligand binding. Additionally, the extent of competition is comparable to the one previously observed for Pry1p from *S*. *cerevisiae* [11]. Hence, Rbe1p and Rbt4p are able to specifically bind cholesterol *in vitro* with micromolar affinity.

To test if Rbe1p and Rbt4p can also bind and export sterols *in vivo*, we expressed *RBE1* and *RBT4* in heme-deficient *S*. *cerevisiae* cells lacking the sterol deacetylase Say1p, as well as the CAP proteins Pry1p and Pry2p. These mutant cells are able to take up exogenous cholesterol under aerobic conditions due to their heme deficiency, while the deletion of *SAY1* and *PRY1*/*PRY2* leads to intracellular accumulation and blocked secretion of the acetylated cholesterol [7, 18]. Sterol export in the different strains can be quantified by labeling the cells with [^14^C]-cholesterol and analyzing the ratio of intra- and extracellular cholesteryl acetate by thin layer chromatography [7]. Confirming the results of the *in vitro* binding assay, expression of either *RBE1* or *RBT4* in the *S*. *cerevisiae pry1Δpry2Δ* mutant could rescue the block in cholesteryl acetate export (Fig 2C). Quantification of export rates revealed that expression of *RBE1* or *RBT4* resulted in a significantly higher export index compared to the *pry1Δ pry2Δ* mutant, indicating that lipid export might be a conserved function of fungal CAP proteins (Fig 2D).

**Fig 3:**
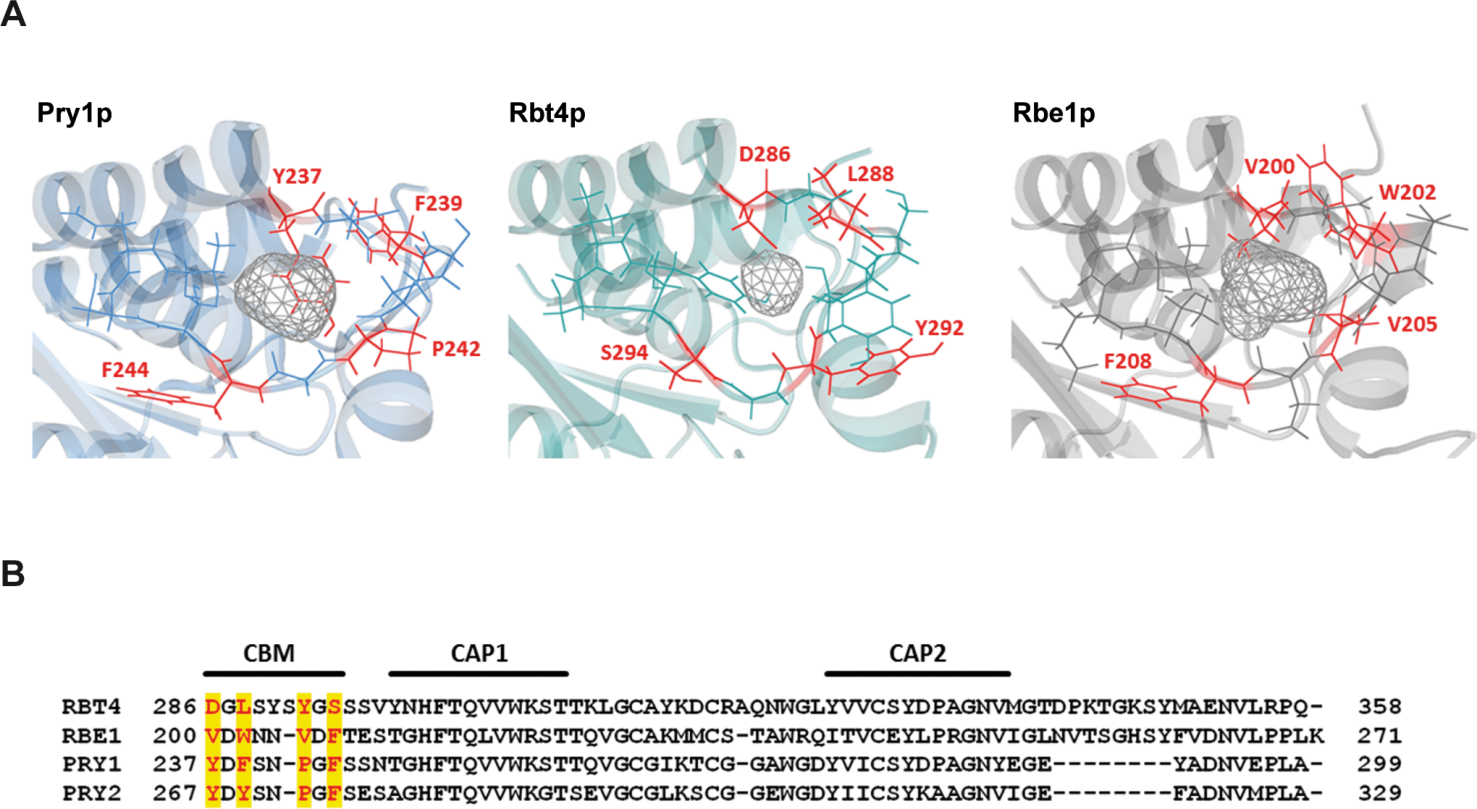
Sequential and structural comparison between different sterol binding domains. **(A)** 3D model showing the C-terminus of Rbe1p, Rbt4p and Pry1p. Homology models for Rbe1p and Rbt4p were created using the web-based I-Tasser server, while the Pry1p structure represents PBD entry 5ete. Residues forming the cholesterol binding cavity are shown as sticks and CBM residues are additionally colored in red. **(B)** Multiple sequence alignment containing the C-terminal residues, including the CBM and CAP1/CAP2 motifs, of Rbe1p (*C*. *albicans*), Rbt4p (*C*. *albicans*), Pry1p (*S*. *cerevisiae*) and Pry2p (*S*. *cerevisiae*). Residues of the CBM are shown in red.

Despite this functional complementation, sequence similarity in the caveolin-binding motif (CBM) is rather low between Rbe1p, Rbt4p and Pry1p. Some aromatic residues shown to be important for sterol binding of Pry1p [11] are conserved in the Rbe1p primary sequence (W202, F208), while none of the residues is conserved in Rbt4p (Fig 3B). Nevertheless, 3D homology modeling of Rbe1p and Rbt4p showed that the CBM region of both proteins forms surface accessible cavity structures, which are a prerequisite for sterol-binding [30] (Fig 3A).

Sterol binding and export function of *S*. *cerevisiae* Pry1p and Pry2p has been linked to lipid proofreading and detoxification of small hydrophobic compounds like eugenol, a phenolic compound present in plant essential oils [7]. However, in contrast to these findings, we could not observe hypersensitivity of the *C*. *albicans rbe1Δrbt4Δ* mutant in the presence of high eugenol concentrations (S6 Fig).

## Discussion

Members of the CAP protein superfamily have emerged as novel virulence factors in fungi, but remain a poorly characterized class of proteins [31, 32]. To expand this knowledge, we analyzed the localization and functional properties of the CAP proteins Rbe1p and Rbt4p from *C*. *albicans*.

Our analysis revealed that Rbe1p can be attached to the yeast cell wall via disulfide bonds, while Rbt4p is constitutively secreted under the experimental conditions used. This cell wall association of Rbe1p was strongly dependent on yeast cell growth. In line with our results, different mass spectrometric analyses of the *C*. *albicans* yeast cell surface proteome have identified peptides corresponding to Rbe1p [33, 34]. A study by Hernaez *et al*. used a combination of trypsin and 5 mM DTT to extract and digest proteins located at the outer layer of the yeast cell wall before doing mass spectrometry. Comparing the detected peptides after tryptic digest with and without DTT, they interestingly only identified Rbe1 peptides in DTT-treated cells, also indicating a disulfide-bond dependent localization of Rbe1p. Despite these results, Rbe1p was mainly considered an extracellular protein and the observed cell wall association was interpreted as an intermediate of the secretory pathway.

The precise mode of disulfide-dependent attachment is still unknown and could be mediated by direct disulfide bond formation or protein-protein interaction. We also cannot fully exclude the possibility that DTT-treatment might release non-covalently bound Rbe1p due to increased cell wall permeability [35].

As there are significant amounts of Rbe1p localized to the cell wall under yeast growth conditions, it might be plausible to assume that this fraction is of physiological relevance. Rbe1p might play a role in cell wall-associated processes, as Ene *et al*. found that a *Δrbe1* strain grown on lactate was more susceptible to cell wall-associated stresses like calcofluor white or high osmolality [36]. However, there is no evidence for a direct function of Rbe1p during cell wall construction or remodeling [3]. The exclusive localization of Rbe1p at chitin-rich budding sites of *C*. *albicans* yeast cells might also indicate a role in immune evasion, as the exposure β-1-3 glucan and chitin at these sites activates host pattern recognition receptors [37, 38]. In agreement with enhanced killing of the *Δrbe1Δrbt4* strain by neutrophils [3], Rbe1p might be involved in masking PAMPs, affecting fungal recognition by phagocytes, but this needs to be validated in the future.

A key finding of our work is that Rbe1p and Rbt4p can bind cholesterol *in vitro* and functionally complement the sterol export defect observed in the *S*. *cerevisiae pry1Δpry2Δ* mutant *in vivo*. The affinity for cholesterol (K_d_ Rbe1~5.78 μM; K_d_ Rbt4 ~3.3 μM) is lower than the one observed for Pry proteins in *S*. *cerevisiae* (K_d_ Pry1 ~ 0.7 μM; K_d_ Pry2 ~ 0.6 μM), but still in the micromolar range, as reported for other cholesterol-binding proteins [30, 39, 40]. In agreement with the observation that cholesterol-binding sites in soluble proteins always adopt cavity or pocket-like structures to minimize solvent exposure [30], *in silico* homology modeling showed that the CBM regions of Rbe1p and Rbt4p are able to form surface accessible cavities.

Based on our observations, we hypothesize that the sterol binding and export properties of Rbe1p and Rbt4p primarily affect the extracellular milieu of *C*. *albicans*, rather than, intracellular sterol homeostasis or sterol detoxification processes. Proper sterol distribution is required for hyphal formation in *C*. *albicans* [41], but as the deletion of *RBE1* and *RBT4* has no influence on morphology [3], both genes seem to be negligible in this context. In a competitive growth assay using a collection of barcoded heterozygous *C*. *albicans* deletion mutants, *RBT4* was shown to affect the resistance against a synthetic ergosterol derivative [42]. This finding might be in line with the observed sterol binding and export properties of Rbt4p, but this phenotype has not been sufficiently validated using a homozygous mutant or the appropriate revertant strain to draw a conclusion at this point. Binding or sequestering of host sterols might therefore be a better aspect trying to explain the virulence phenotype associated with *RBE1* and *RBT4* deletion. In accordance with our hypothesis, cholesterol binding has been described as an important feature of microbial virulence factors [43] and phagocytic neutrophils, which are linked to the virulence phenotype of Rbe1p and Rbt4p, depend on a proper distribution of cholesterol for adhesion, polarization and NADPH oxidase activation [44].

## Supporting information

S1 Fig Glycosylation of Rbe1p and Rbt4p. Supernatant (S) or DTT-sensitive cell wall fraction (P) wa analyzed for the presence of V5-tagged Rbe1p and Rbt4p after enzymatic removal of N-glycan with PNGase F or chemical removal of O-glycan using β-elimination. Before deglycosylation, samples were dialysed against H_2_O to remove interfering components, and then treated either with 2,000 units PNGase F (New England Biolabs) for 1 h at 37°C or with the *GlycoProfile™ ß-Elimination Kit* (Sigma-Aldrich) for 14 h at 4°C. After deglycosylation, samples were directly separated by SDS-PAGE and detected by immunoblotting with a mouse anti-V5 antibody.
(TIF)

S2 Fig Comparison between intra- and extracellular levels of Rbe1p and Rbt4p in *C*. *albicans*. Supernatant (S) and cytoplasmic lysate (L) from *C*. *albicans* yeast and hyphal cells were analyzed for the presence of V5-tagged Rbe1p or Rbt4p. The corresponding strains were grown in SC-medium at 30°C (yeast) or RPMI-medium at 37°C (hyphae) for 5 h, the supernatant was concentrated using ultracentrifugation and the cells were lysed using bead beating. An equal relative volume of each fraction was loaded in each lane of an SDS-PAGE and the proteins were detected by immunoblotting with a mouse anti-V5 antibody.
(TIF)

S3 Fig Purification and identification of the Rbe1p homodimer. **(A)** Enrichment of the V5-tagged Rbe1p monomer and dimer after V5 affinity purification using the DTT-sensitive cell wall fraction extracted from the V5-tagged *C*. *albicans* strain and the untagged control strain. Elution fractions were separated by SDS-PAGE, stained with Coomassie Brilliant Blue G-250 and the indicated bands at ~ 75 kDa (monomer) and ~ 135 kDa (dimer), including the corresponding bands from the control, were excised from the gel and subsequently analyzed by Nano-LC-MS/MS. **(B)** Comparison of the proteins identified by mass spectrometry in the 135 kDa band (dimer) of the Rbe1-V5 sample versus the corresponding band of the control sample. Only proteins having a unique peptide count > 1 in either sample are shown. For the complete list see S4 Table.
(TIF)

S4 Fig Localization of Rbe1p and Rbt4p in *C*. *albicans* yeast and hyphal cells. Wild type *C*. *albicans* (WT, SC5314) and strains expressing V5-tagged Rbe1p or Rbt4p were stained against the V5-epitope using indirect immunofluorescence. To induce hyphal growth, an overnight culture or the corresponding strains was inoculated in fresh RPMI-medium and grown for 5 h at 37°C. For yeast growth, cells were diluted in SC-medium and grown for 5 h at 30°C. Formaldehyde fixed cells were stained using a primary mouse anti-V5 antibody and a secondary Alexa Fluor 555 coupled goat anti-mouse antibody (orange). Note that laser exposure times were adjusted according to the strength of the fluorescence signal; 200 ms for yeast Rbe1-V5, all other images 1.500 ms. Scale bar: 20 μm.
(TIF)

S5 Fig Comparison of N-acetyl-glucosamine localization and deposition in *C*. *albicans* wild type cells and *Δrbe1* cells. Live *C*. *albicans* wild type cells or *Δrbe1* cells grown in SC medium at 30°C were stained with FITC-conjugated wheat germ agglutinin (green) as described in the Methods section and imaged by fluorescence microscopy. Scale bar: 20 μm.
(TIF)

S6 Fig Influence of *RBE1* and *RBT4* deletion on the growth of *C*. *albicans* in the presence of the plant oil eugenol. Cells of the indicated genotype were serially diluted 10-fold and spotted onto YPD plates containing or lacking eugenol. Plates were incubated at 30°C overnight.
(TIF)

S7 Fig Original and codon optimized *RBE1* **(A)** and *RBT4* **(B)** sequences used for heterologous expression in *E*. *coli*. Modified codons are shown in red.
(PDF)

S1 Table Strains used in this study. Name, genotype, parental strain and source of the strains used in this study.
(PDF)

S2 Table Primers used in this study. Restriction sites are underlined.
(PDF)

S3 Table Plasmids created in this study. (PDF)

S4 Table Proteins identified by mass spectrometry. Peptide counts for the proteins identified in the monomer and dimer gel band of the Rbe1p-V5 sample and the control sample.
(PDF)
