# Supplementary figures and images for: Localization and functional characterization of the pathogenesis-related proteins Rbe1p and Rbt4p in Candida albicans
Source: PLoS One. 2018 Aug 6;13(8):e0201932. doi: 10.1371/journal.pone.0201932 (PMC6078311; doi:10.1371/journal.pone.0201932)

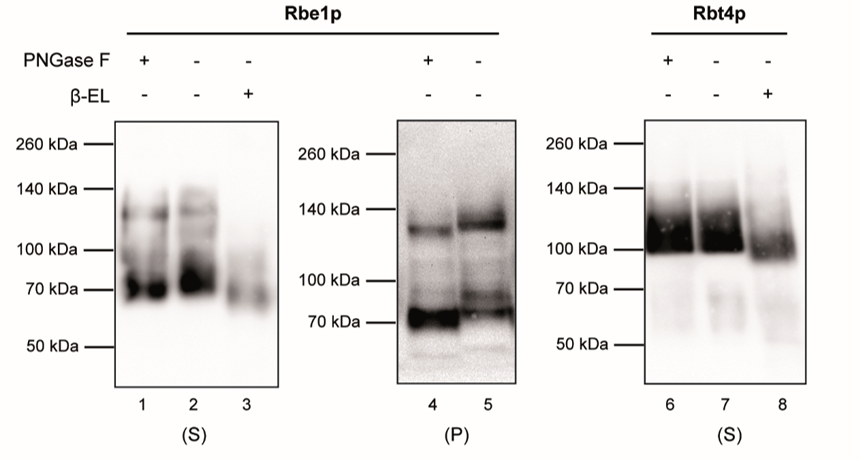

Supplement: S1 Fig — Supernatant (S) or DTT-sensitive cell wall fraction (P) wa analyzed for the presence of V5-tagged Rbe1p and Rbt4p after enzymatic removal of N-glycan with PNGase F or chemical removal of O-glycan using β-elimination. Before deglycosylation, samples were dialysed against H2O to remove interfering components, and then treated either with 2,000 units PNGase F (New England Biolabs) for 1 h at 37°C or with the GlycoProfile™ ß-Elimination Kit (Sigma-Aldrich) for 14 h at 4°C. After deglycosylation, samples were directly separated by SDS-PAGE and detected by immunoblotting with a mouse anti-V5 antibody. (TIF) [file pone.0201932.s001.tif]

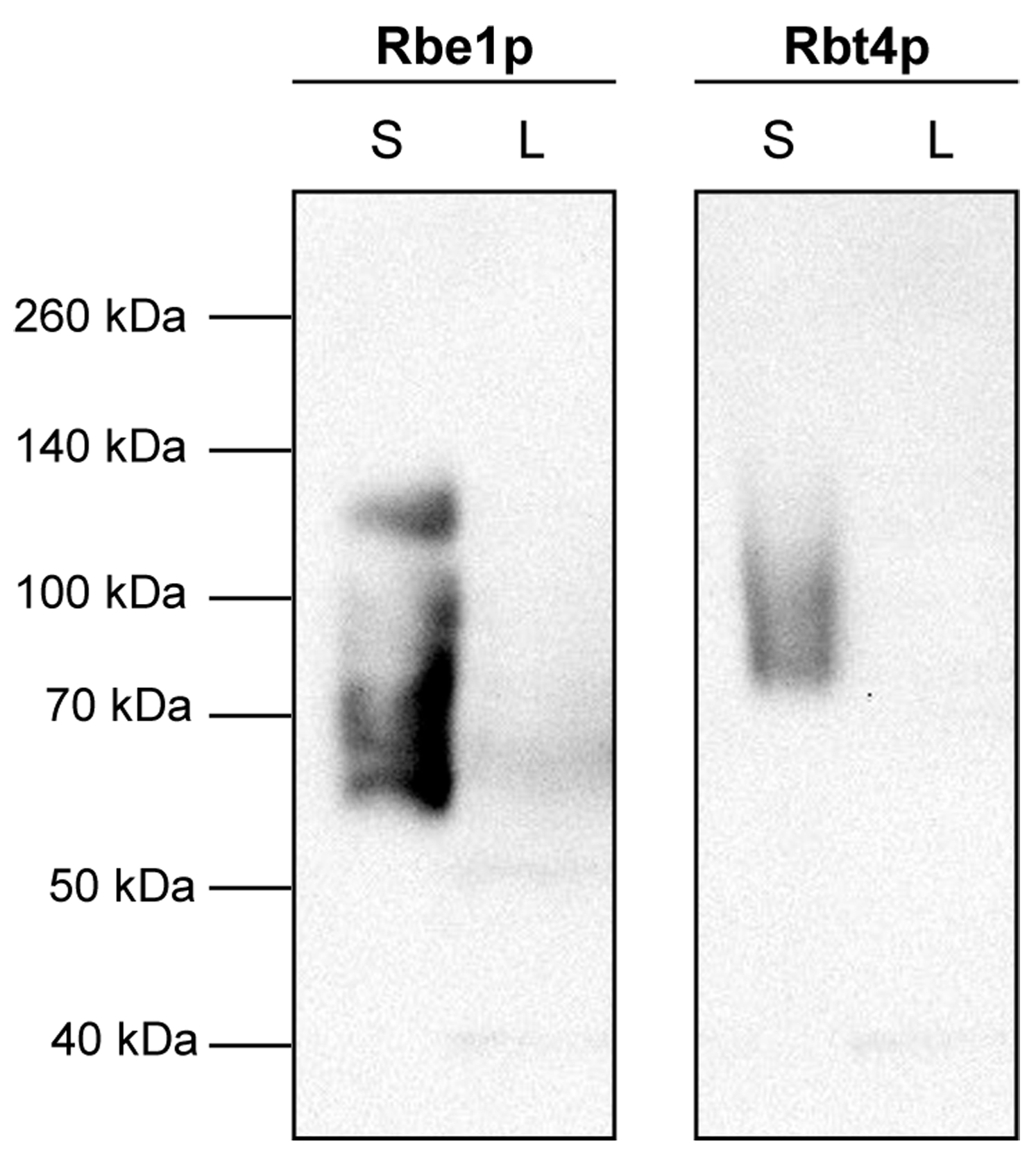

Supplement: S2 Fig — Supernatant (S) and cytoplasmic lysate (L) from C. albicans yeast and hyphal cells were analyzed for the presence of V5-tagged Rbe1p or Rbt4p. The corresponding strains were grown in SC-medium at 30°C (yeast) or RPMI-medium at 37°C (hyphae) for 5 h, the supernatant was concentrated using ultracentrifugation and the cells were lysed using bead beating. An equal relative volume of each fraction was loaded in each lane of an SDS-PAGE and the proteins were detected by immunoblotting with a mouse anti-V5 antibody. (TIF) [file pone.0201932.s002.tif]

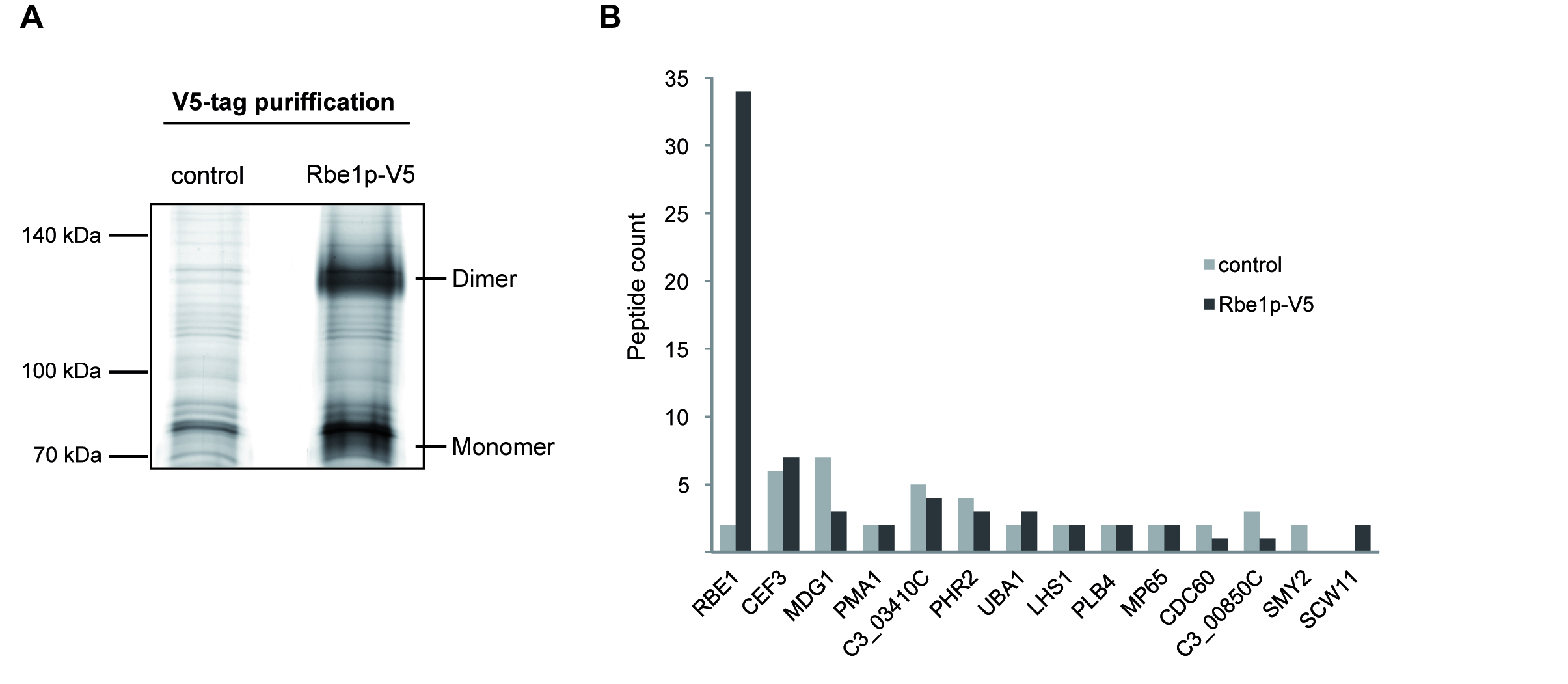

Supplement: S3 Fig — (A) Enrichment of the V5-tagged Rbe1p monomer and dimer after V5 affinity purification using the DTT-sensitive cell wall fraction extracted from the V5-tagged C. albicans strain and the untagged control strain. Elution fractions were separated by SDS-PAGE, stained with Coomassie Brilliant Blue G-250 and the indicated bands at ~ 75 kDa (monomer) and ~ 135 kDa (dimer), including the corresponding bands from the control, were excised from the gel and subsequently analyzed by Nano-LC-MS/MS. (B) Comparison of the proteins identified by mass spectrometry in the 135 kDa band (dimer) of the Rbe1-V5 sample versus the corresponding band of the control sample. Only proteins having a unique peptide count > 1 in either sample are shown. For the complete list see S4 Table. (TIF) [file pone.0201932.s003.tif]

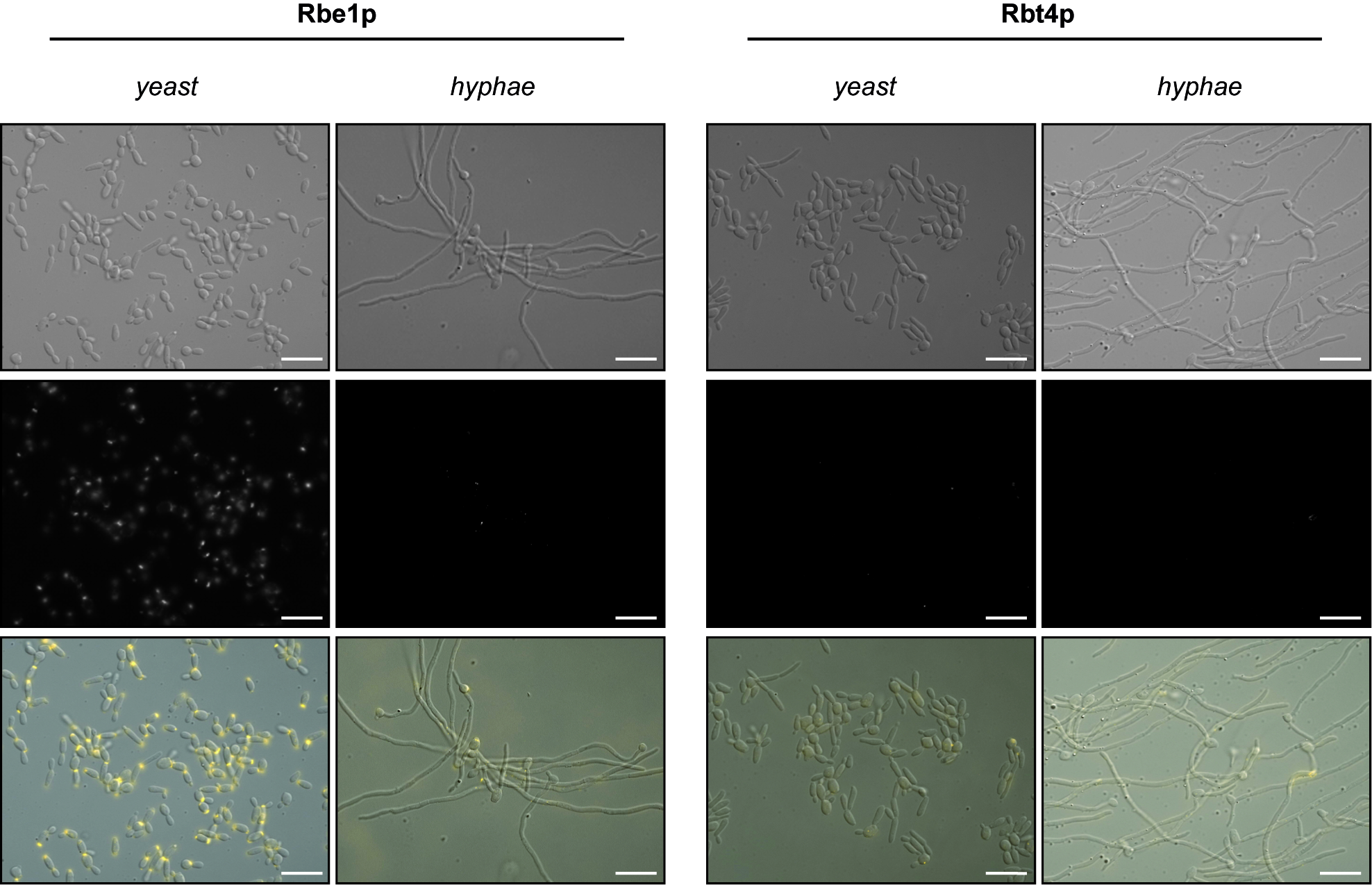

Supplement: S4 Fig — Wild type C. albicans (WT, SC5314) and strains expressing V5-tagged Rbe1p or Rbt4p were stained against the V5-epitope using indirect immunofluorescence. To induce hyphal growth, an overnight culture or the corresponding strains was inoculated in fresh RPMI-medium and grown for 5 h at 37°C. For yeast growth, cells were diluted in SC-medium and grown for 5 h at 30°C. Formaldehyde fixed cells were stained using a primary mouse anti-V5 antibody and a secondary Alexa Fluor 555 coupled goat anti-mouse antibody (orange). Note that laser exposure times were adjusted according to the strength of the fluorescence signal; 200 ms for yeast Rbe1-V5, all other images 1.500 ms. Scale bar: 20 μm. (TIF) [file pone.0201932.s004.tif]

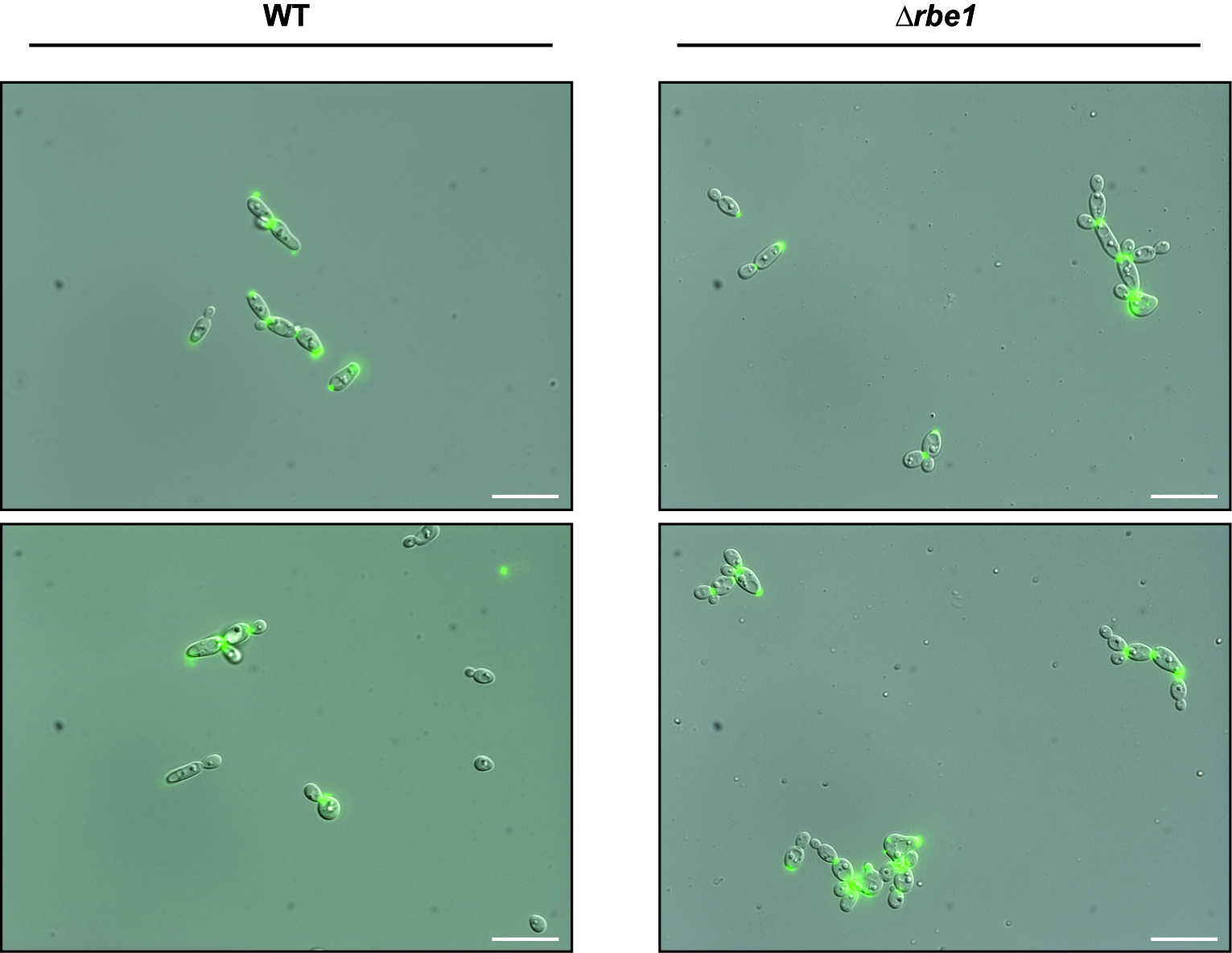

Supplement: S5 Fig — Live C. albicans wild type cells or Δrbe1 cells grown in SC medium at 30°C were stained with FITC-conjugated wheat germ agglutinin (green) as described in the Methods section and imaged by fluorescence microscopy. Scale bar: 20 μm. (TIF) [file pone.0201932.s005.tif]

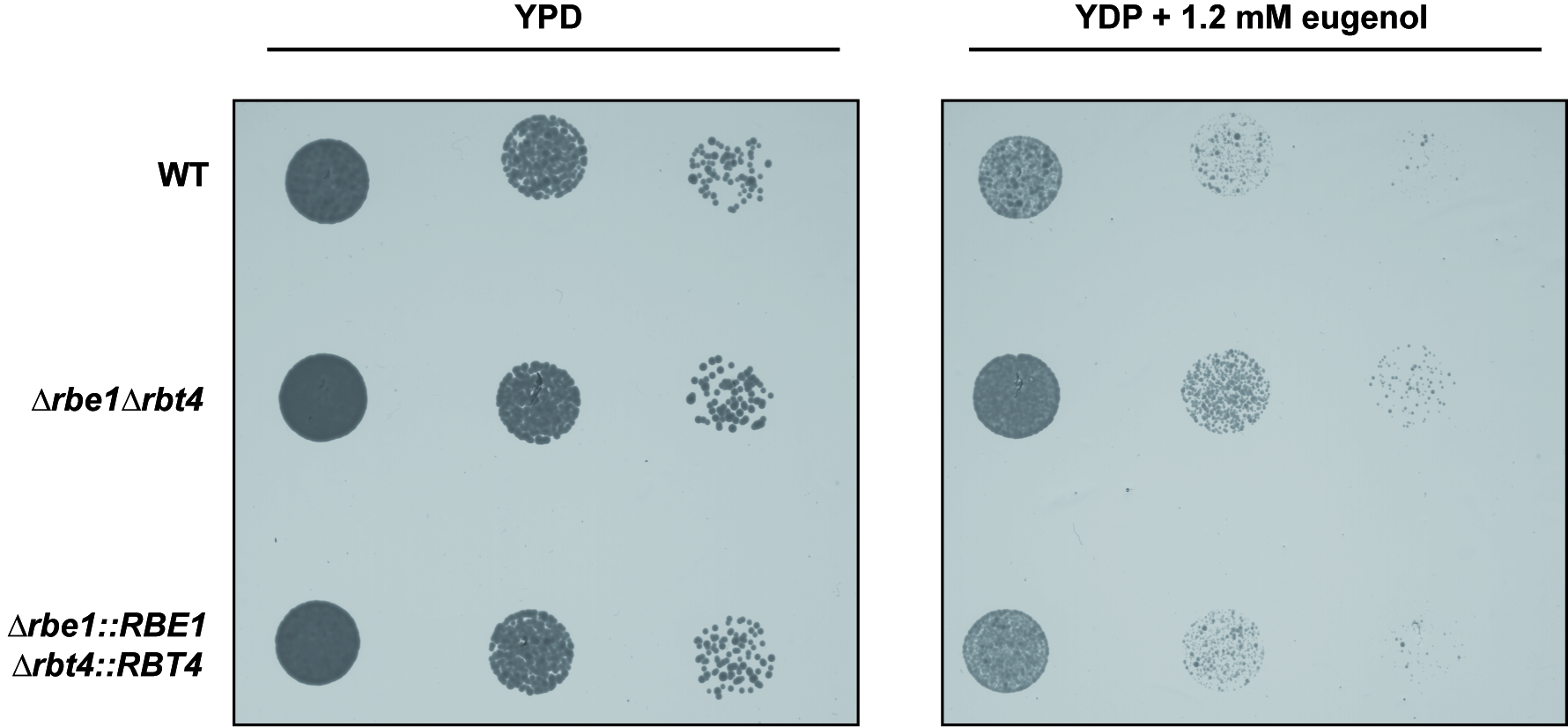

Supplement: S6 Fig — Cells of the indicated genotype were serially diluted 10-fold and spotted onto YPD plates containing or lacking eugenol. Plates were incubated at 30°C overnight. (TIF) [file pone.0201932.s006.tif]
