# Supplementary material for: Localization and functional characterization of the pathogenesis-related proteins Rbe1p and Rbt4p in Candida albicans
Source: PLoS One. 2018 Aug 6;13(8):e0201932. doi: 10.1371/journal.pone.0201932 (PMC6078311; doi:10.1371/journal.pone.0201932)
Supplement: S7 Fig — Original and codon optimized RBE1 (A) and RBT4 (B) sequences used for heterologous expression in E. coli. Modified codons are shown in red. (PDF) [file pone.0201932.s007.pdf]

A

**Rbe1 Optimized**	ATGAAGATTACCAATACCCTGCTGAATGCGGCGGCGCTGCTGGCGGTTACCGAAGCGGCG
**Rbe1 Original**	ATGAAGATAACAAATACTTTACTTAATGCTGCTGCATTATTAGCAGTCACAGAAGCTGCC
**Rbe1 Optimized**	ACCATCACCAAGTTTTTCACCGCGAGCACCCAGACCCTGTTCGTGACCCAGACCAGCCAA
**Rbe1 Original**	ACTATCACCAAATTCTTTACTGCCTCAACTCAAACTCTTTTTGTCACCCAAACTAGTCAA
**Rbe1 Optimized**	ACCGTGGTTGCGACCAAGAGCTTTGTTGAGACCATCTACAGCGCTCCGCCGAAGCAGCTG
**Original**	ACCGTTGTGGCCACCAAATCATTTGTTGAAACAATTTATTCAGCTCCACCAAAACAACTC
**Rbe1 Optimized**	ACCAGCAAAACCCAAGACAGCACCAGCCCGACCACCAGCAGCGTGAACAGCCTGACCAGC
**Rbe1 Original**	ACTAGTAAAACTCAAGACTCTACCTCCCCAACTACTAGCTCCGTCAACTCATTAACCAGT
**Rbe1 Optimized**	AGCAGCGCGACCAGCTATGTGGAGACCACCACCCCGGCGCCGAGCAGCAGCACCCTGACC
**Rbe1 Original**	AGTTCGGCCACTTCATATGTCGAAACTACAACTCCAGCTCCATCTTCGTCAACTTTGACA
**Rbe1 Optimized**	ACCAGCACCATCAGCAGCAGCACCGCGAGCGAAGACAGCGATGCGACCCCGACCGCGGAT
**Rbe1 Original**	ACTTCCACTATATCTTCATCTACTGCTAGTGAAGATTCAGACGCTACACCTACCGCCGAT
**Rbe1 Optimized**	GTGGAGTTTGCGGAGGAAATTCTGAAGGAACACAACGTGAAACGTGCGCTGCATGGTGTT
**Rbe1 Original**	GTTGAATTTGCCGAGGAAATTTTGAAAGAACATAATGTCAAGAGAGCACTCCATGGTGTG
**Rbe1 Optimized**	CCGGCGCTGAGCTGGAGCAACAAGCTGGCGGAGTACGCGCAGGACTATGCGAACACCGGC
**Rbe1 Original**	CCTGCACTTAGTTGGAGTAACAAATTAGCCGAATACGCTCAAGATTACGCTAACACTGGA
**Rbe1 Optimized**	TTTGATTGCAGCAACCTGAACCTGAAACACAGCGGTGGCCCGTATGGTGAAAACCTGGCG
**Rbe1 Original**	TTCGATTGTAGCAATCTTAACTTGAAACATTCTGGTGGTCCATACGGTGAAAATTTGGCT
**Rbe1 Optimized**	GCGGGTTATATGGGTGGCATCAGCCCGGTTGACGCGTGGTACGATGAGATTAGCATGGTG
**Rbe1 Original**	GCTGGTTATATGGGTGGAATTAGTCCTGTTGATGCTTGGTATGATGAAATCTCCATGGTT
**Rbe1 Optimized**	GACTGGAACAACGTTGATTTCACCGAAAGCACCGGTCACTTTACCCAGCTGGTGTGGCGT
**Rbe1 Original**	GATTGGAACAATGTCGATTTCACTGAGTCTACCGGACACTTTACTCAACTTGTATGGCGT
**Rbe1 Optimized**	AGCACCACCCAAGTTGGCTGCGCGAAAATGATGTGCAGCACCGCGTGGCGTCAAATCACC
**Rbe1 Original**	AGTACCACTCAAGTTGGTTGTGCTAAAATGATGTGTTCTACTGCCTGGAGACAAATCACT
**Rbe1 Optimized**	GTGTGCGAATATCTGCCGCGTGGTAACGTTATTGGCCTGAATGTTACCAGCGGTCATAGC
**Rbe1 Original**	GTTTGTGAATACTTGCCTCGTGGTAATGTCATTGGTTTGAATGTCACTTCAGGACACTCA
**Rbe1 Optimized**	TACTTCGTGGACAATGTTCTGCCGCCGCTGAAATAA
**Rbe1 Original**	TACTTTGTTGACAACGTCTTGCCACCTTTAAAGTAA

B

**Rbt4 Optimized**	ATGAAGTTTAGCCAGGTTGCGACCACCGCGGCGATTTTTGCGGGCCTGACCACCGCGGAG
**Rbt4 Original**	ATGAAGTTTTCTCAAGTTGCCACTACTGCTGCCATTTTTGCTGGTTTAACCACTGCTGAA
**Rbt4 Optimized**	ATTGCGTACGTGACCCAGACCCGTGGCGTGACCGTTGGTGAAACCGCGACCGTGGCGACC
**Rbt4 Original**	ATCGCCTATGTCACCCAGACTCGTGGTGTTACTGTTGGTGAAACTGCCACCGTTGCTACA
**Rbt4 Optimized**	ACCGTGACCGTTGGTGCGACCGTTACCGGTGGCGGTCAGGGTCAAGATCAAGTGCAGCAA
**Rbt4 Original**	ACTGTTACCGTTGGTGCAACTGTCACAGGTGGTGGCCAAGGTCAAGATCAAGTTCAACAA
**Rbt4 Optimized**	AGCGCGGCGCCGGAGGCGGATGATATCCAGCAAAGCGCGGTTCCGGAAGCGGACGATATT
**Rbt4 Original**	TCAGCTGCTCCAGAAGCTGATGATATCCAACAATCAGCTGTTCCAGAAGCTGATGATATC
**Rbt4 Optimized**	CAGCAAAGCGTGGTTCCGGAAGCGGAGCCGACCGCGGATGCGGATGGCGGTAACGGTATC
**Rbt4 Original**	CAACAATCAGTTGTTCCAGAAGCTGAACCCACTGCCGATGCTGATGGTGGTAATGGTATT
**Rbt4 Optimized**	GCGATTACCGAGGTGTTCACCACCACCATCATGGGCCAAGAAATTGTGTATAGCGGCGTT
**Rbt4 Original**	GCAATTACCGAAGTCTTTACCACTACCATTATGGGTCAAGAGATTGTTTATTCCGGTGTT
**Rbt4 Optimized**	TACTATAGCTACGGCGAGGAACACACCTATGGCGACGTGCAAGTTCAGACCCTGACCATC
**Rbt4 Original**	TATTACAGTTATGGTGAAGAACATACCTATGGAGACGTTCAAGTTCAAACCCTCACTATT
**Rbt4 Optimized**	GGCGGTGGCGGTTTTCCGAGCGACGATCAGTACCCGACCACCGAGGTTTCTGCGGAAGCG
**Rbt4 Original**	GGGGGTGGCGGCTTCCCTTCAGATGACCAATATCCTACAACTGAAGTTTCTGCTGAGGCT
**Rbt4 Optimized**	AGCCCGAGCGCGGTGACCACCAGCAGCGCGGTTGCGACCCCGGATGCGAAGGTGCCGGAC
**Rbt4 Original**	AGTCCATCTGCTGTTACTACTTCTTCTGCTGTTGCTACTCCTGACGCCAAAGTCCCAGAC
**Rbt4 Optimized**	AGCACCAAAGATGCGAGCCAACCGGCGGCGACCACCGCGAGCGGTAGCAGCAGCGGTAGC
**Rbt4 Original**	TCTACTAAAGACGCTTCTCAACCCGCTGCTACTACAGCTAGTGGCTCCTCTTCTGGTAGT
**Rbt4 Optimized**	AACGACTTCAGCGGTGTTAAGGATACCAAATTTGCGCAGCAAATTCTGGACGCGCACAAC
**Rbt4 Original**	AATGACTTTAGTGGTGTTAAAGATACCAAATTTGCTCAACAAATTTTGGATGCTCACAAC
**Rbt4 Optimized**	CAGAAGCGTGCGCGTCATGGTGTGCCGGACCTGACCTGGGATGCGACCGTTTACCAATAT
**Rbt4 Original**	CAAAAACGTGCTAGACATGGTGTTCCAGATTTGACTTGGGATGCTACTGTTTACCAATAT
**Rbt4 Optimized**	GCGCAGAAGTTCGCGGATCAATACAGCTGCAGCGGTAACCTGCAGCACAGCGGCGGTAAA
**Rbt4 Original**	GCTCAAAAATTTGCTGATCAATACAGTTGTTCTGGTAACTTGCAACACTCTGGTGGTAAA
**Rbt4 Optimized**	TACGGCGAAAACCTGGCGGTTGGCTATGCGGATGGTGCGGCGGCGCTGCAGGCGTGGTAC
**Rbt4 Original**	TATGGTGAAAACTTGGCTGTTGGTTATGCTGATGGTGCTGCTGCTCTTCAAGCTTGGTAC
**Rbt4 Optimized**	GAGGAAGCGGGCAAGGATGGTCTGAGCTACAGCTATGGTAGCAGCAGCGTGTATAACCAC
**Rbt4 Original**	GAAGAAGCTGGTAAGGACGGATTAAGCTACAGTTACGGTTCTTCTTCTGTATACAACCAT
**Rbt4 Optimized**	TTTACCCAAGTGGTTTGGAAGAGCACCACCAAACTGGGCTGCGCGTACAAAGACTGCCGT
**Rbt4 Original**	TTCACTCAAGTTGTCTGGAAATCAACCACCAAACTTGGTTGTGCTTACAAGGATTGTCGT
**Rbt4 Optimized**	GCGCAGAACTGGGGTCTGTACGTGGTTTGCAGCTATGATCCGGCGGGCAACGTGATGGGC
**Rbt4 Original**	GCTCAAAACTGGGGATTATACGTTGTTTGTTCCTACGACCCAGCTGGTAACGTTATGGGT
**Rbt4 Optimized**	ACCGACCCGAAGACCGGCAAGAGCTATATGGCGGAAAATGTTCTGCGTCCGCAATAA
**Rbt4 Original**	ACTGATCCAAAGACTGGTAAATCTTATATGGCTGAAAATGTCTTGAGACCACAATAA
