# Supplementary material for: Localization and functional characterization of the pathogenesis-related proteins Rbe1p and Rbt4p in Candida albicans
Source: PLoS One. 2018 Aug 6;13(8):e0201932. doi: 10.1371/journal.pone.0201932 (PMC6078311; doi:10.1371/journal.pone.0201932)
Supplement: S1 Table — Name, genotype, parental strain and source of the strains used in this study. (PDF) [file pone.0201932.s008.pdf]

**Strain**	**Genotype**	**Parental strain**	**Reference**
SC5314	*Candida albicans* wild type (clinical isolate)		[1]
Rbe1-CT-V5/His6	*RBE1*/*rbe1*Δ::FRT *RBE1* V5 His6	SC5314	This study
Rbt4-CT-V5/His6	*RBT4*/*rbt4*Δ::FRT *RBT4* V5 His6	SC5314	This study
rbe1Δ	*rbe1Δ*::FRT/*rbe1Δ*::FRT	SC5314	[2]
rbe1Δrbt4Δ	*rbt4Δ*::FRT/*rbt4Δ*::FRT<br>*rbe1Δ*::FRT/*rbe1Δ*::FRT	SC5314	[2]
say1Δhem1Δ	*MATα his3Δ1 leu2Δ0 ura3Δ0 lys2Δ0*<br><br>*say1::KanMX4 hem1::LEU2*		[3]
pry1Δpry2<br><br>*Δsay1Δhem1Δ*	*MATα his3Δ1 leu2Δ0 ura3Δ0 lys2Δ0*<br><br>*pry1::KanMX4 pry2::URA3 say1::HIS3 hem1::LEU2*		[4]

1. Gillum AM, Tsay EY, Kirsch DR (1984) Isolation of the Candida albicans gene for orotidine-5'-phosphate decarboxylase by complementation of S. cerevisiae ura3 and E. coli pyrF mutations. Mol Gen Genet 198: 179-182.

2. Rohm M, Lindemann E, Hiller E, Ermert D, Lemuth K, et al. (2013) A family of secreted pathogenesis-related proteins in Candida albicans. Mol Microbiol 87: 132-151.

3. Tiwari R, Köffel R, Schneiter R (2007) An acetylation/deacetylation cycle controls the export of sterols and steroids from S. cerevisiae. The EMBO Journal 26: 5109-5119.

4. Choudhary V, Schneiter R (2012) Pathogen-Related Yeast (PRY) proteins and members of the CAP superfamily are secreted sterol-binding proteins. Proc Natl Acad Sci U S A 109: 16882-16887.
