# Supplementary material for: Localization and functional characterization of the pathogenesis-related proteins Rbe1p and Rbt4p in Candida albicans
Source: PLoS One. 2018 Aug 6;13(8):e0201932. doi: 10.1371/journal.pone.0201932 (PMC6078311; doi:10.1371/journal.pone.0201932)
Supplement: S2 Table — Restriction sites are underlined. (PDF) [file pone.0201932.s009.pdf]

Construct	Primer Name	Sequence 5´- 3´
**pSFS2A CT Rbe1 FR1 ORF FR2**	RBE1 FR1 for ApaI	GCTCGGGCCCATGCCACAAGTCTCCCATTC
	RBE1 ORF wo TAA rev XhoI	CCGCTCGAGCTTTAAAGGTGGCAAGACGTT
	RBE1 FR2 for NotI	ATAAGAATGCGGCCGCAAAAAAAAAGAAAGATTAATTTGATGATG
	RBE1 FR2 rev SacII	TCCCCGCGGGTGAGTACCACCACCACCAA
**pSFS2A CT Rbt4 FR1 ORF FR2**	RBT4 FR1 for ApaI	GCTCGGGCCCCAATTCACGCATTCCAACTG
	RBT4 ORF wo TAA rev XhoI	CCGCTCGAGTTGTGGTCTCAAGACATTTTCAG
	RBT4 FR2 for NotI	ATAAGAATGCGGCCGCATGATTTATTAGACCTAGTCCTTGTCCA
	RBT4 FR2 rev SacI	CGAGCTCAGATCAGGAAGCTCGGGAAT
**pRS416 Rbe1 ORF w/o signal sequence**	Alpha factor Rbe1 for	AGAAGGGGTATCTTTGGATAAAAGAGAGGCTGAAGCCACTATCACCAAATTCTTTACT
	Alpha factor Rbe1 rev	CAAAGGAAAAGGGGCCTGTCTCGAGGTCGACGGTATCGATTTACTTTAAAGGTGGCAAGACG
**pRS416 Rbt4 ORF w/o signal sequence**	Alpha factor Rbt4 for	AGAAGGGGTATCTTTGGATAAAAGAGAGGCTGAAGAAATCGCCTATGTCACCCA
	Alpha factor Rbt4 rev	CAAAGGAAAAGGGGCCTGTCTCGAGGTCGACGGTATCGATTTATTGTGGTCTCAAGACATTTTC
