# Supplementary material for: Localization and functional characterization of the pathogenesis-related proteins Rbe1p and Rbt4p in Candida albicans
Source: PLoS One. 2018 Aug 6;13(8):e0201932. doi: 10.1371/journal.pone.0201932 (PMC6078311; doi:10.1371/journal.pone.0201932)
Supplement: S3 Table — (PDF) [file pone.0201932.s010.pdf]

Plasmid	Created from plasmid	Source
pSFS2A CT-V5/His6		[1]
pSFS2A CT-V5/His6 Rbe1 FR1	pSFS2A CT	This study
pSFS2A CT-V5/His6 Rbe1 FR1 ORF FR2	pSFS2A CT Rbe1 FR1	This study
pSFS2A CT-V5/His6 Rbt4 FR1	pSFS2A CT	This study
pSFS2A CT-V5/His6 Rbt4 FR1 ORF FR2	pSFS2A CT Rbe1 FR1	This study
pET-19b(+) NT-His10 Rbe1 opt	pET-19b(+)	GenScript
pET-19b(+) NT-His10 Rbt4 opt	pET-19b(+)	GenScript
pRS416 Rbe1 ORF	pRS416	This study
pRS416 Rbt4 ORF	pRS416	This study

1. Palzer S, Bantel Y, Kazenwadel F, Berg M, Rupp S, et al. (2013) An expanded genetic code in Candida albicans to study protein-protein interactions in vivo. Eukaryot Cell 12: 816-827.
