# Supplementary material for: Localization and functional characterization of the pathogenesis-related proteins Rbe1p and Rbt4p in Candida albicans
Source: PLoS One. 2018 Aug 6;13(8):e0201932. doi: 10.1371/journal.pone.0201932 (PMC6078311; doi:10.1371/journal.pone.0201932)
Supplement: S4 Table — Peptide counts for the proteins identified in the monomer and dimer gel band of the Rbe1p-V5 sample and the control sample. (PDF) [file pone.0201932.s011.pdf]

Identified Proteins	Accession Number (NCBI)	Monomer sample		Dimer sample	
		Control	Rbe1-V5	Control	Rbe1-V5
hypothetical protein MGQ_01364 [Candida albicans P76067] Repressed by Efg1 (*RBE1*)	KHC42070.1	0	17	2	34
likely HSP70 family chaperonin [Candida albicans SC5314]	XP_713714.1	7	14	0	0
chaperone DnaK [Candida albicans 19F]	KGU15991.1	9	11	0	0
polyadenylate-binding protein, cytoplasmic and nuclear [Candida albicans P94015]	KGQ95663.1	2	1	0	0
translation elongation factor 3 [Candida albicans]	CAA78282.1	0	0	6	7
NADH-ubiquinone oxidoreductase 75 kDa subunit [Candida albicans WO-1]	EEQ44016.1	5	12	0	0
hypothetical protein W5Q_01381 [Candida albicans SC5314]	KHC84156.1	0	0	7	3
leucyl-tRNA synthetase [Candida albicans WO-1]	EEQ43286.1	0	0	2	1
acetate--CoA ligase [Candida albicans SC5314]	AOW26103.1	0	2	0	0
coatomer protein complex, subunit alpha (xenin) [Candida albicans P37005]	KGQ92106.1	0	0	2	1
transketolase [Candida albicans WO-1]	EEQ42384.1	1	4	0	0
pyruvate carboxylase [Candida albicans P94015]	KGQ86632.1	0	0	1	0
chaperone protein CaMsi3p [Candida albicans]	BAB71816.1	2	0	0	0
hypothetical protein MEU_02536 [Candida albicans P37005]	KGQ92022.1	0	0	3	1
plasma membrane ATPase [Candida albicans WO-1]	EEQ44146.1	0	0	2	2
hypothetical protein MG1_02805 [Candida albicans GC75]	KGQ98165.1	0	0	5	4

protein EPD1 precursor [Candida albicans WO-1]	EEQ43106.1	0	0	4	3
ubiquitin-activating enzyme E1 [Candida albicans P94015]	KGQ86919.1	0	0	2	3
hypothetical protein MEO_00975 [Candida albicans P94015]	KGQ90660.1	2	3	0	0
hypoxia up-regulated 1 [Candida albicans P94015]	KGQ88838.1	0	0	2	2
hypothetical protein CAWG_03911 [Candida albicans WO-1]	EEQ45582.1	0	0	2	2
hypothetical protein MEU_00181 [Candida albicans P37005]	KGQ98590.1	1	3	0	0
hypothetical protein MG3_02408 [Candida albicans P78048]	KGR13974.1	0	0	2	2
conserved hypothetical protein [Candida albicans WO-1]	EEQ45027.1	0	0	0	1
hypothetical protein MEO_03840 [Candida albicans P94015]	KGQ85522.1	0	0	2	0
hypothetical protein CAALFM_C200360CA [Candida albicans SC5314]	AOW27090.1	2	0	0	0
cytoskeleton assembly control protein Sla2p [Candida albicans]	CAA08750.1	0	0	1	1
flavocytochrome c [Candida albicans P37005]	KGQ97513.1	1	2	0	0
valyl-tRNA synthetase, mitochondrial precursor [Candida albicans WO-1]	EEQ46054.1	0	0	1	1
glycerol-3-phosphate dehydrogenase, mitochondrial [Candida albicans GC75]	KGQ92654.1	2	0	0	0
ATP-dependent RNA helicase DED1 [Candida albicans P94015]	KGQ88221.1	1	0	0	0
drug resistance protein 2 [Candida albicans]	AAB96797.1	0	0	0	1
hypothetical protein CAWG_05603 [Candida albicans WO-1]	EEQ47048.1	1	0	0	0
conserved hypothetical protein [Candida albicans WO-1]	EEQ46430.1	0	0	0	2
conserved hypothetical protein [Candida albicans WO-1]	EEQ42790.1	1	0	0	0

heat shock protein SSC1, mitochondrial precursor [Candida albicans WO-1]	EEQ47268.1	2	0	0	0
vacuolar ATP synthase subunit a [Candida albicans WO-1]	EEQ44988.1	0	1	0	0
